# Heat transfer analysis of the forced air quenching with non-isothermal and non-uniform oxidation

**DOI:** 10.1371/journal.pone.0253240

**Published:** 2021-06-17

**Authors:** Yue Zhang, Jian Yang, Ming-Xin Gao, Hua Sono

**Affiliations:** 1 School of Mechanical Engineering and Automation, University of Science and Technology Liaoning, Anshan, China; 2 School of Materials and Metallurgy, University of Science and Technology Liaoning, Anshan, China; 3 Engineering Training Center, University of Science and Technology Liaoning, Anshan, China; Tongji University, CHINA

## Abstract

In this paper, the heat transfer characteristics of the forced air quenching with non-isothermal and non-uniform oxidation are investigated. By introducing the variations of interfacial temperature and oxygen partial pressure, a three-layered non-isothermal high-temperature oxidation kinetic model is developed, in which a discrete-time modeling method is employed to solve the problem of integration of the transient terms, and a special interfacial grid treatment is used for considering the growth of each oxide layer and updating of the thermal properties. Moreover, a parameter identification method using the multi-objective genetic algorithm is proposed for the inverse solution of the oxidation parabolic parameters based on the measured scale thicknesses in oxidation experiment. A case study of the forced air quenching of a Q235 disk is presented to validate the availability of the developed formulas. Then the interfacial heat transfer characteristics are analyzed, while the numerical solutions with and without oxidation are both performed for in-depth comparison. Results indicate that the active quenching region is mainly centralized in the vicinity of stagnation region. The radial variation regularity of the temperature difference across the total oxide layer is mainly determined by the thermal conductivity and the scale thickness. The existence of the oxide scale actually produces a certain thermal resistance during the quenching process and the effects of the oxide scale increases with the radial coordinate due to the interfacial temperature distribution. The results obtained can provide theoretical derivation for precise control of the internal phase transformation during the forced air quenching process.

## Introduction

With the advantages of green quenchant and good uniformity, the forced air quenching achieved by the Laval nozzle and compressed air is widely used in the heat treatments of steel rail, steel slag and aluminum alloy [1–3]. As an open heat treatment process, it is inevitable to generate oxidizing reaction on the surface of the high-temperature workpiece, while the formed composite oxide scale with high thermal resistance would have important impact on heat transfer of the forced air quenching for quality control [4–7]. A typical thin oxide scale is normally a three-layered structure composed of hematite, magnetite and wustite from the inside out. Since the variations of the quenching temperature and oxygen partial pressure at different positions are quite different, the scale thickness and physical parameters of each oxide layer are constantly and non-uniformly changing during the forced air quenching process. Moreover, given the complicated multi-layered high-temperature oxidation mechanism as well as the inherent strong shock wave, large variable gradient and wide-range Mach number, the heat transfer analysis of the forced air quenching with considering the oxidation reaction still remains a great deal of difficulty and challenge.

In the actual quenching process, when the high-speed airflow strongly impacts on the high-temperature quenching surface, the intense oxidation reaction will occur on the fluid-solid interface due to the interactive transportation of the oxygen anions and metal cations which indicates a diffusion-controlled oxidation process. So far, many oxidation models of practical significance have been well developed to describe the diffusion-controlled high temperature oxidation mechanism, including approximately two patterns, namely the isothermal and the non-isothermal oxidation kinetic models. For isothermal oxidation, the most classic ones are the various parabolic models [8–11], such as the TPB parabolic model and the Wagner parabolic model. However, compared with the isothermal oxidation, the oxide-scale growth characteristics under non-isothermal conditions are more complex and may be completely different. To deal with the non-isothermal conditions, an improved oxidation kinetic model [12] has been proposed by generally integrating the linear, parabolic and cubic oxidation laws. Meanwhile, extensive researches have also been focused on the non-isothermal oxidation behaviors of SiC Whiskers, hot-rolled steel, fuel samples, Al_4_SiC_4_ and TiC powders while abundant achievements have been accumulated [13–17]. Despite this, the most common practice for predicting the non-isothermal oxidation is to derive the parabolic parameters from the corresponding subdivision isothermal experiments [18]. However, due to the particularity of the forced air quenching conditions, mainly including the severe convection between the high-speed airflow and high-temperature quenching surface, the short-time sharp change in temperature and the varied oxygen partial pressures and cooling rates at different positions, it is rather difficult for the existing non-isothermal oxidation kinetic models to deal with the high-temperature oxidation behavior sufficiently during the forced air quenching. More importantly, it is extremely difficult to simulate the open quenching conditions of the forced air quenching for the traditional isothermal or non-isothermal oxidation experiment which is implemented in a closed environment. And it is almost impossible to derive the corresponding parabolic parameters of each oxide layer from the isothermal experiments or even the non-isothermal experiments under these real complex quenching conditions. Hence, it is of great significance to develop a new non-isothermal oxidation kinetic model and corresponding effective identification method for its parabolic parameters.

In addition, the introduction of the high-temperature oxidation generally results in a multi-layer composite structure which is, from the inside out, specifically divided into the metal substrate, wustite, magnetite, hematite and airflow. For the past few years, the heat transfer problems in multi-layer composite structures have attracted considerable attention [19]. For example, Shen et al. [20] analyzed the heat transfer characteristics between the high-speed airflow and the gap-cavity-gap structure based on a simplified quasi-steady method. Rahman et al. [21] investigated the heat transfer coefficient at the solid-fluid conjugate interface of a composite trapezoidal channel during magnetic heating. Zhang et al. [22] studied the heterogeneous two-dimensional conjugate heat transfer in synthetic polymers by considering the interactions between the fillers and the base materials. Gao et al. [23] carried out the heat transfer analyses in a two-layer solid medium and an infinite composite solid by an improved lattice Boltzmann method. The above researches provide important references for the analysis of heat transfer of the forced air quenching. As the multi-layered oxide scale with high thermal resistance has important impact on the heat transfer of the forced air quenching, the introduction of the high-temperature oxidation is of great necessity and significance. However, to the author’s knowledge, there has been no effective heat transfer model of the forced air quenching to deal with the growth characteristics of the multi-layered oxide scale contributed by the multi-layered non-isothermal and non-uniform high-temperature oxidation at the conjugate interface.

In order to introduce the high-temperature oxidation behavior during the quenching process, a three-layered non-isothermal high-temperature oxidation kinetic model is developed by considering the variations of interfacial temperature and oxygen partial pressure along the radial direction, in which a discrete-time modeling method is employed to solve the problem of integration of the transient terms. To cope with the high-temperature oxidation, a special grid treatment is provided for considering the growth of each oxide layer and updating of the thermal properties. As an indispensable part of the oxidation kinetic model, a parameter identification method using the multi-objective genetic algorithm is proposed for the inverse solution of the oxidation parabolic parameters based on the measured scale thicknesses in oxidation experiment. Furthermore, a two-dimensional axisymmetric finite-volume model of heat transfer of the forced air quenching with considering the non-isothermal and non-uniform high-temperature oxidation is established, while a segregated fluid-solid coupling procedure is employed to improve the efficiency of the whole solution to the heat transfer problem. To verify the validity of the developed model, both the numerical and experimental examples of the forced air quenching of a Q235 disk are carried out in present work. On this basis, the heat transfer characteristics of the forced air quenching are studied under the conditions with and without the high-temperature oxidation. Additionally, the interaction mechanism between the heat transfer and high-temperature oxidation is further analyzed.

## Physical and numerical model

### Geometry description

**Fig 1:**
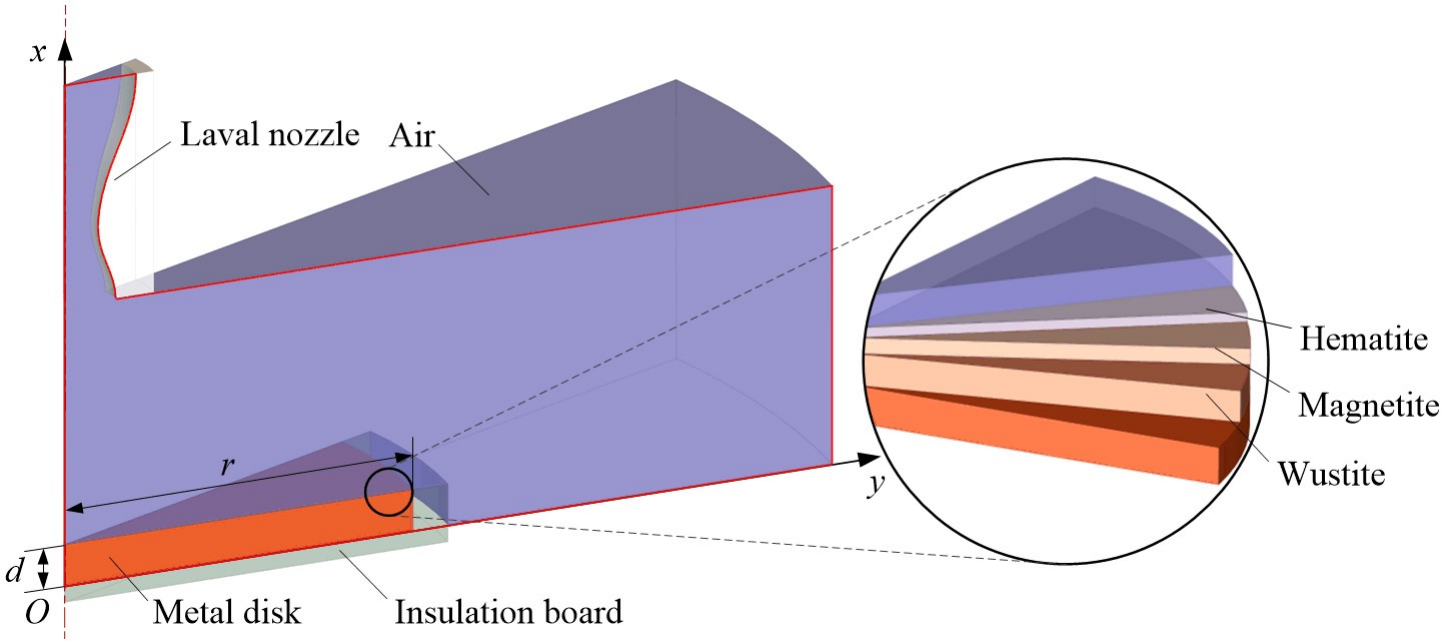
Schematic diagram of the geometry of the forced air quenching of a metal disk.

The axisymmetric geometry of the forced air quenching of a *r*×*d* metal disk is shown in Fig 1.

Generally, the oxide scale structures of steel during short-term high-temperature oxidation in air are similar to those of pure iron which contain a thick inner wustite layer, a thin middle magnetite layer and an ultra-thin outer hematite layer [18, 24]. Therefore, the classical three-layered geometric structure of the oxide scale is taken into consideration at the conjugate interface between the cryogenic supersonic airflow and the high-temperature metal disk (see Fig 1). To construct the insulation boundary conditions for the metal disk, the insulation board is introduced in present work. And due to the limited effects on the heat transfer of the forced air quenching, the geometry of the Laval nozzle is not considered in the computational domain marked with solid red lines.

### Governing equations

To perform the non-isothermal and non-uniform high-temperature oxidation more efficiently, a segregated fluid-solid coupling procedure is employed to calculate the conjugate heat transfer of the forced air quenching. The core idea is to consider the flow field as steady-state while treat the temperature fields of the fluid domain and solid domain as transient. The general two-dimensional axisymmetric governing equations for steady-state flow of the forced air quenching in the orthogonal curvilinear coordinates (*ξ*, *η*) can be written as [25]

$$\frac{\partial }{\partial \xi }[\rho U\phi -\frac{\Gamma_{\phi }}{J}(\alpha \frac{\partial \phi }{\partial \xi }-\beta \frac{\partial \phi }{\partial \eta })]+\frac{\partial }{\partial \eta }[\rho V\phi -\frac{\Gamma_{\phi }}{J}(\gamma \frac{\partial \phi }{\partial \xi }-\beta \frac{\partial \phi }{\partial \eta })]=b_{\phi }\frac{\partial p}{\partial \xi }+c_{\phi }\frac{\partial p}{\partial \eta }+JS_{\phi }$$ (1)

where

$$J=x_{\xi }y_{\eta }-x_{\eta }y_{\xi },\;U=uy_{\eta }-vx_{\eta },\;V=vx_{\xi }-uy_{\xi },\alpha =x_{\eta }^{2}+y_{\eta }^{2},\beta =x_{\xi }x_{\eta }+y_{\xi }y_{\eta },\gamma =x_{\xi }^{2}+y_{\xi }^{2}$$ (2)

where *U*, *V* and *ϕ* (1, *u*, *v*, *k*, *ε*, *T*) are the contravariant velocity components and the general variable, respectively. *b*_*ϕ*_ and *c*_*ϕ*_ are the pressure coefficients. *Γ*_*ϕ*_ and *S*_*ϕ*_ are the effective diffusion coefficient and the generalized source term (including the viscous and axisymmetric items), respectively. *α*, *β*, *γ* and *J* are the introduced geometric quantities and the Jacobian of the transformation, respectively. *ρ* and *P* refer the density and pressure, respectively. To close the governing equations, the equation of state for ideal gas is considered

ρ=p/RT (3)

Here *R* denotes the gas constant (*R* = 287.026 J/(kg·K)).

In order to conveniently solve the heat transfer in solid and fluid domains of the forced air quenching in a unified manner, the concept of the pseudo density $\tilde{\rho }=\rho c_{p}$ (*c*_*p*_ refer to the specific heat) is introduced. Then, the general governing equations for the transient fluid-solid conjugate heat transfer in the orthogonal curvilinear coordinates (*ξ*, *η*) can be written as

$$J\frac{\partial (\tilde{\rho }T)}{\partial t}+\frac{\partial }{\partial \xi }[\tilde{\rho }UT-\frac{\Gamma_{T}}{J}(\alpha \frac{\partial T}{\partial \xi }-\beta \frac{\partial T}{\partial \eta })]+\frac{\partial }{\partial \eta }[\tilde{\rho }VT-\frac{\Gamma_{T}}{J}(\gamma \frac{\partial T}{\partial \xi }-\beta \frac{\partial T}{\partial \eta })]=JS_{T}$$ (4)

Owing to its excellent capability in impinging jet simulation, the realizable *k*-*ε* turbulence model [26, 27] is employed to consider the effects of turbulent fluctuation on the heat transfer of the forced air quenching. In order to achieve better convergence in iterations, some appropriate treatments should be taken to avoid the occurrences of negative turbulent kinetic energy and turbulent dissipation rate. To complement the realizable *k*-*ε* turbulence model, the standard wall-function method [28] is adopted to handle the fluid flow and heat transfer in the near-wall region. And the production limiter presented by Menter [29] is considered in the turbulence equations to solve the problem of excessive turbulent kinetic energy produced in the stagnation region.

By introducing the equivalent dynamic viscosity *μ*_eqv_ and the equivalent thermal conductivity *λ*_eqv_, the wall shear stress *τ*_w_ and the wall heat flux *q*_w_, regarding as the additional source items of the momentum and energy equations accordingly, can be expressed as follows

$$\tau_{\text{w}}=\mu_{eqv}({\tilde{u}}_{P}-{\tilde{u}}_{\text{w}})/{\hat{y}}_{P},\;q_{\text{w}}=\lambda_{eqv}(T_{P}-T_{\text{w}})/{\hat{y}}_{P}$$ (5)

where ${\hat{y}}_{P}$ is the normal distance from the wall-adjacent point *P* to the wall. ${\tilde{u}}_{P}$ and *T*_*P*_ represent the resultant velocity and temperature at point *P*, respectively. *T*_w_ is the wall temperature. Based on the wall-function method, the equivalent dynamic viscosity *μ*_eqv_ and the equivalent thermal conductivity *λ*_eqv_ with wall velocity ${\tilde{u}}_{\text{w}}=0$ can be calculated by

$$\mu_{\text{eqv}}=\mu y_{P}^{+}/u_{P}^{+},\;\lambda_{\text{eqv}}=\mu c_{p}y_{P}^{+}/T_{P}^{+}$$ (6)

where *μ* is the dynamic viscosity. $u_{P}^{+}$, $y_{P}^{+}$ and $T_{P}^{+}$ are the dimensionless velocity, distance and temperature at the wall-adjacent point *P*. The equivalent dynamic viscosity *μ*_eqv_ and the equivalent thermal conductivity *λ*_eqv_ will be used to calculate the effective diffusion coefficients of the momentum and energy equations at the wall-adjacent point *P* beyond the boundary layer during the iterations, respectively.

### Boundary conditions

Based on the distribution characteristics of fluid flow of the classical impinging-jet and the actual conditions of the forced air quenching, the far enough outlet of the fluid flow is set as the entrainment boundary, while the total pressure and temperature, the initial velocities, turbulent kinetic energy and turbulent dissipation rate are set for the inlet boundary. As part of the standard wall-function method, the turbulent kinetic energy production and turbulent dissipation rate at the wall-adjacent point *P* is determined by [30, 31]

$$G_{k}=\tau_{\text{w}}^{2}/\kappa \rho C_{\mu }^{1/4}k_{p}^{1/2}{\hat{y}}_{P},\;\varepsilon_{P}=C_{\mu }^{3/4}k_{P}^{3/2}/\kappa {\hat{y}}_{P}$$ (7)

where *κ* and *C*_*μ*_ refer to the von Kármán constant and the turbulence model coefficient, respectively. Additionally, the wall shear stress *τ*_w_ and wall heat flux *q*_w_ obtained by Eq 5 are linearly added into the source items of the momentum and energy equations, respectively. The radial velocity and other flow variable gradients at the axisymmetric boundary are set to be zero. The adiabatic boundary is applied to the bottom of the metal disk and the outside of the insulation board due to the heat insulation treatments. Moreover, the no-slip wall and adiabatic boundary conditions are adopted at the interface of the airflow and Laval nozzle. The pressure outlet with one standard atmospheric pressure is used at the outlet boundary. In order to uniformly solve the temperature fields of the fluid and solid domains, the boundary conditions of equal temperature and equal heat flux at the conjugate interface need to be satisfied. Moreover, the harmonic mean method is suggested for calculating the interface thermal conductivity to further meet the conditions of equal heat flux at the conjugate interface.

### Oxidation kinetic model and its parameter identification method

**Fig 2:**
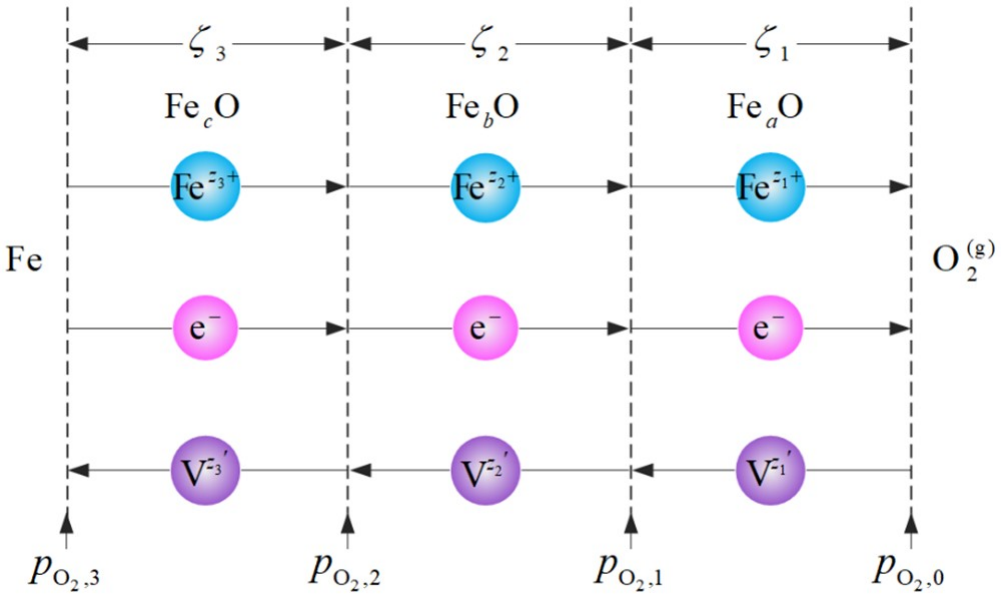
General diffusion model for the growth of a three-layered oxide structure.

For the high-temperature oxidation would have a great influence on heat transfer of the forced air quenching [4–6], a three-layered non-isothermal oxidation kinetic model is proposed by considering the variations of the temperature and oxygen partial pressure along the radial direction. Just as with other oxidation models [32], it is also assumed that the growth rates of the three oxide scales are controlled by diffusion, the outward diffusion of cations is greater than the inward diffusion of anions, the cationic flux is independent of the diffusion distance, the electronic conduction is dominant and the phase boundary is in equilibrium. The general diffusion model for the growth of a three-layered oxide structure of the forced air quenching is shown in Fig 2.

For the short-time high-temperature oxidation of steel is similar to that of pure iron, the total oxidation reaction based on the Wagner’s theory [33–35] can be generally written as the following form

$$(aA+bB+cC)\text{Fe}+[\left(A+B+C\right)/2]{\text{O}}_{2}\to A{\text{Fe}}_{a}\text{O}+B{\text{Fe}}_{b}\text{O}+C{\text{Fe}}_{c}\text{O}$$ (8)

where *a* = 2/3, *b* = 3/4, and *c* = 1. *A*, *B* and *C* refer to the molar partitioning fractions. By assuming the cation vacancies in Fe_2_O_3_, Fe_3_O_4_ and FeO to be ${\text{V}}_{\text{Fe}}^{z_{1}{}^{\prime }}$, ${\text{V}}_{\text{Fe}}^{z_{2}{}^{\prime }}$ and ${\text{V}}_{\text{Fe}}^{z_{3}{}^{\prime }}$, respectively, the phase consumption and cation diffusion at the FeO/Metal substrate interface can be given by

$$(aA+bB+cC)\text{Fe+}(aA+bB+cC){\text{V}}_{\text{Fe}}^{z_{3}{}^{\prime }}=(aA+bB+cC){\text{Fe}}^{z_{3}+}+z_{3}(aA+bB+cC){\text{e}}^{-}$$ (9)

At the Fe_3_O_4_/FeO interface, the growth of the FeO layer depends on the consumption of Fe_3_O_4_ and partial cations, the corresponding reaction can be expressed as follows

$$(c-b)C{\text{Fe}}^{z_{3}+}+C{\text{Fe}}_{b}\text{O}+z_{3}(c-b)C{\text{e}}^{-}=C{\text{Fe}}_{c}\text{O+}(c-b)C{\text{V}}_{\text{Fe}}^{z_{3}{}^{\prime }}$$ (10)

and the number of the remaining cations is (*aA* + *bB* + *cC*) − (*c* − *b*)*C* = *aA* + *bB* + *bC*. Similarly, the reactions at the Fe_2_O_3_/ Fe_3_O_4_ interface can be represented as follows

$$(b-a)(B+C){\text{Fe}}^{z_{2}+}+(B+C){\text{Fe}}_{a}\text{O}+z_{2}(b-a)(B+C){\text{e}}^{-}=(B+C){\text{Fe}}_{b}\text{O+}(b-a)(B+C){\text{V}}_{\text{Fe}}^{z_{2}{}^{\prime }}$$ (11)

then the number of the remaining cations is (*aA* + *bB* + *bC*) − (*b* − *a*)(*B* + *C*) = *a*(*A* + *B* + *C*). Finally, at the Air/Fe_2_O_3_ interface, the remaining cations will react with the oxygen in the cryogenic supersonic airflow, given by

$$a(A+B+C){\text{Fe}}^{z_{1}+}+[\left(A+B+C\right)/2]{\text{O}}_{2}+z_{1}a(A+B+C){\text{e}}^{-}=(A+B+C){\text{Fe}}_{a}\text{O+}a(A+B+C){\text{V}}_{\text{Fe}}^{z_{1}{}^{\prime }}$$ (12)

According to the numbers of the consumed and remaining cations indicated in above reactions at each oxidation interface, the rates of thickening of the three oxide layers Fe_2_O_3_, Fe_3_O_4_ and FeO can be, respectively, written as [36]

$$\frac{\text{d}\zeta_{l}}{\text{d}t}=\frac{{\tilde{\varpi }}_{l}{\bar{J}}_{l}{\bar{V}}_{l}-}{d_{l}}\frac{\text{d}\zeta_{l+1}}{\text{d}t}\cdot \frac{{\bar{V}}_{l}}{{\bar{V}}_{l+1}},\;l=1,2,3$$ (13)

$${\tilde{\varpi }}_{1}=1,\;{\tilde{\varpi }}_{2}=\frac{B+C}{aA+bB+bC},\;{\tilde{\varpi }}_{3}=\frac{C}{aA+bB+cC},\;d_{l}=\frac{l^{2}}{12}-\frac{l}{6}+\frac{3}{4}$$ (14)

where *d*_1_ = *a*, *d*_2_ = *b* and *d*_3_ = *c*. *ζ*_*l*_ is the instantaneous thickness of *l*th oxide layer. ${\bar{J}}_{l}$ and ${\bar{V}}_{l}$ are the uniform molar flux and the average molar volume of *l*th oxide layer, respectively. *ζ*_4_ and ${\bar{V}}_{4}$ in Eq 13 denote any nonzero constant. In accordance with the total cationic fluxes of the three oxide layers indicated in Eqs 8–12, the following relationships can be obtained

$$\frac{{\bar{J}}_{2}}{{\bar{J}}_{3}}=\frac{aA+bB+bC}{aA+bB+cC},\;\frac{{\bar{J}}_{1}}{{\bar{J}}_{2}}=\frac{aA+aB+aC}{aA+bB+bC}$$ (15)

Substituting Eq 15 into Eq 13 and applying the following fundamental equations [36]

$${\bar{J}}_{l}{\bar{V}}_{l}/d_{l}=K_{l}/\zeta_{l},\;l=1,2,3$$ (16)

yields

$$\text{d}\zeta_{l}/\text{d}t={\hat{\varpi }}_{l}K_{l}/\zeta_{l}$$ (17)

$${\hat{\varpi }}_{1}=\frac{aA}{aA+bB+cC},\;{\hat{\varpi }}_{2}=\frac{bB}{bA+bB+cC},\;{\hat{\varpi }}_{3}=\frac{cA+cC}{cA+cB+cC}$$ (18)

where *K*_*l*_ is the parabolic rate for the diffusion-controlled growth of *l*th oxide layer, given by

$$K_{l}=\frac{1}{2d_{l}}\int_{p_{{\text{O}}_{2},l}^{}}^{p_{{\text{O}}_{2},l-1}^{}}D_{l}\text{d}(\text{ln}p_{{\text{O}}_{2}}),\;l=1,2,3$$ (19)

where $p_{{\text{O}}_{2}}$ is the oxygen partial pressure. *D*_*l*_ is the self-diffusion function, defined by

$$D_{l}=F_{l}\text{exp}(-E_{l}/\tilde{R}T),\;l=1,2,3$$ (20)

where $\tilde{R}$ refers to the molar gas constant ($\tilde{R}=8.314$ J/(mol·K)). *F*_*l*_ and *E*_*l*_ represent the frequency factor and the diffusion activation energy of *l*th oxide layer, respectively. It is worth noting that the oxide layer of FeO will stop growing and do not consume the cations when the temperature is below 843.15 K. So, in that case, the parabolic rate of the FeO layer should be set to zero. For the cooling rate and pressure of the airflow presents obvious non-uniform distribution at the conjugate interface, the oxygen partial pressures at each oxide interface vary widely in both axial and radial directions of the three-layered oxide scale. At the Air/Fe_2_O_3_ conjugate interface, the oxygen partial pressure is directly determined by its volume fraction and the fluid pressure *p*_conj_ as follows

$$p_{{\text{O}}_{2},0}=V_{\text{f}}p_{\text{conj}}/p_{\text{op}}$$ (21)

where *V*_f_ is the volume fraction of oxygen in the airflow (*V*_f_ = 21%). *p*_op_ refers to the operating pressure (*p*_op_ = 101325 Pa). The oxygen partial pressures at other three interfaces (Fe_2_O_3_/Fe_3_O_4_, Fe_3_O_4_/FeO and FeO/Metal substrate) under normal pressure can be determined, respectively, by

$$\begin{array}{l} p_{{\text{O}}_{2},1}=\text{exp}(-25.08985+0.01920T)\\ p_{{\text{O}}_{2},2}=\text{exp}(-38.00354+0.02468T)\\ p_{{\text{O}}_{2},3}=\text{exp}(-35.94221+0.02057T) \end{array}$$ (22)

As the temperature is time dependent, the self-diffusion functions and oxygen partial pressures are all functions of time, which further results that the oxidation kinetic model of the forced air quenching can not be directly obtained by integrating Eq 17 over time. In order to solve this problem, a discrete-time modeling method is employed in which the time *t* is discretized into a finite number of time step Δ*t*. Thus, in each time step, the self-diffusion functions and oxygen partial pressures can be considered as constant. Integrating Eq 17 over time in a time step that from *t* to *t* + Δ*t* one can obtain

$$\zeta_{l,t+\Delta t}^{2}-\zeta_{l,t}^{2}=2{\hat{\varpi }}_{l}K_{l,t}\Delta t,\;l=1,2,3$$ (23)

further yields

$$\zeta_{l,t}^{2}-\zeta_{l,0}^{2}=2\Delta t{\hat{\varpi }}_{l}\sum_{k=1}^{m}K_{l,t}$$ (24)

$$\zeta_{l}^{2}=\varpi_{l}\Delta t\sum_{k=1}^{m}\text{ln}(p_{{\text{O}}_{2},l-1,k}^{}/p_{{\text{O}}_{2},l,k}^{})F_{l}\text{exp}(-E_{l}/\tilde{R}T_{k})+\zeta_{l,0}^{2}$$ (25)

$$\varpi_{1}=\frac{A}{aA+bB+cC},\;\varpi_{2}=\frac{B}{bA+bB+cC},\;\varpi_{3}=\frac{A+C}{cA+cB+cC}$$ (26)

where *ζ*_*j*,0_ is the initial thickness at time zero. *m* is the number of time step. Moreover, the whole instantaneous thickness of the three oxide layers can be expressed by

$$\xi_{t}=\sum_{l=1}^{3}\sqrt{\varpi_{l}\Delta t\sum_{k=1}^{m}\text{ln}(p_{{\text{O}}_{2},l-1,k}^{}/p_{{\text{O}}_{2},l,k}^{})F_{l}\text{exp}(-E_{l}/\tilde{R}T_{k})+\zeta_{l,0}^{2}}$$ (27)

Due to the influence of the strong impact of the high-speed airflow on the quenching surface, the non-uniformly varied oxygen partial pressures, cooling rates and temperatures will be formed at the multi-layered heat transfer interfaces. Under these complex and ever-changing quenching conditions, it is almost impossible to derive the undetermined parabolic parameters from the traditional isothermal or non-isothermal oxidation experiment. Moreover, it is extremely difficult to simulate the open quenching conditions of the forced air quenching for the traditional isothermal or non-isothermal oxidation experiment which is implemented in a closed environment. In order to effectively address the above problems, an inverse solution method in combination with the multi-objective optimization using non-dominated sorting genetic algorithm (NSGA-II) [37] is proposed to identify the corresponding parabolic parameters of the developed oxidation kinetic model, based on the actual oxidation experiment of the forced air quenching. Because of the low efficiency of linear weight for Pareto points, the solution of the multi-objective optimization problems still generally depends on the evolutionary algorithm. The NSGA-II (Non-dominated sorting genetic algorithm) [37] is the very famous and frequently used genetic evolutionary algorithm for solving the multi-objective optimization problems, therefore, is employed to identify the parabolic parameters. The multi-objective optimization formulation with weighting coefficients can be defined by

$$\begin{array}{ll} \text{min} & F_{l}=[f_{1}(E_{l},F_{l}),f_{2}(E_{l},F_{l})]\\ \text{s}.\text{t}. & 1\times 10^{-3}\leq E_{l}\leq 1\times 10^{3},\;1\times 10^{3}\leq F_{l}\leq 1\times 10^{6},\;l=1,2,3 \end{array}$$ (28)

where

$$\begin{array}{l} f_{1}(E_{l},F_{l})=\sqrt{w_{1}{\left(\zeta_{l}-\zeta_{l}^{*}\right)}_{y=0}^{2}+w_{4}{\left(\zeta_{l}-\zeta_{l}^{*}\right)}_{y=60}^{2}}\\ f_{2}(E_{l},F_{l})=\sqrt{w_{2}{\left(\zeta_{l}-\zeta_{l}^{*}\right)}_{y=0}^{2}+w_{3}{\left(\zeta_{l}-\zeta_{l}^{*}\right)}_{y=60}^{2}} \end{array}$$ (29)

where $\zeta_{l}^{*}$ is the experimental scale thickness of *l*th oxide layer. *n* is the number of the implemented oxidation experiment. *w*_1_, *w*_2_, *w*_3_ and *w*_4_ refer to the weighting coefficients. *ζ*_*l*_ is the numerical instantaneous thickness of *l*th layer of oxide scale corresponding to $\zeta_{l}^{*}$ at the same position and quenching time. Moreover, it is worth mentioning that, during the short-time oxidation of the forced air quenching, the interfaces between the three oxide layers are hard to distinguish when the total scale thickness is in micron level. Thus, in order to get adequate data to calculate the values of *E*_*l*_ and *F*_*l*_ from the total scale thickness measured in the oxidation experiments, the observed proportions of 1%, 4% and 95% for the instantaneous thicknesses of Fe_2_O_3_, Fe_3_O_4_ and FeO layers during the long-time oxidation [38] are employed for the multi-objective optimization.

Additionally, to achieve the optimum solution from a set of Pareto-optimal points in the above multi-objective optimization problem, an evaluation function with weighting coefficients is defined as the following

$$h\left(F_{l}\right)=\sqrt{{\tilde{w}}_{1}f_{1}{\left(E_{l},F_{l}\right)}^{2}+{\tilde{w}}_{2}f_{2}{\left(E_{l},F_{l}\right)}^{2}},\;l=1,2,3$$ (30)

where ${\tilde{w}}_{1}$ and ${\tilde{w}}_{2}$ denote the weighting coefficients corresponding to the defined objective functions *f*_1_ and *f*_2_, respectively.

### Discretization and solution of governing equations

The governing equations are discretized by the finite volume method. Suppose that *i* − 1/2, *i* + 1/2, *j* − 1/2 and *j* + 1/2 are the west, east, south and north cell faces of point (*i*, *j*), respectively. Then, with Δ*ξ* = Δ*η* = 1, the discretizations of Eqs 1 and 4 can be obtained as follows

$${\left[\rho U\phi -\alpha \frac{\Gamma_{\phi }}{J}\frac{\partial \phi }{\partial \xi }\right]}_{i-1/2}^{i+1/2}+{\left[\rho V\phi -\gamma \frac{\Gamma_{\phi }}{J}\frac{\partial \phi }{\partial \xi }\right]}_{j-1/2}^{j+1/2}=b_{\phi }(p_{i+1/2}-p_{i-1/2})+c_{\phi }(p_{j+1/2}-p_{j-1/2})+JS_{\phi }+S_{\phi }^{\text{non}}$$ (31)

$$J\frac{{\left(\tilde{\rho }T\right)}_{i,j}}{\Delta t}+{\left[\tilde{\rho }UT-\alpha \frac{\Gamma_{T}}{J}\frac{\partial T}{\partial \xi }\right]}_{i-1}^{i+1}+{\left[\tilde{\rho }VT-\gamma \frac{\Gamma_{T}}{J}\frac{\partial T}{\partial \xi }\right]}_{j-1}^{j+1}=JS_{T}+S_{T}^{\text{non}}+J\frac{{\left(\tilde{\rho }T\right)}_{i,j}^{*}}{\Delta t}$$ (32)

where $S_{\phi }^{\text{non}}(\phi =u,v,k,\varepsilon ,T)$ refers to the source item contributed by the nonorthogonality, given by

$$S_{\phi }^{\text{non}}=-{\left[\beta \frac{\Gamma_{\phi }}{J}\frac{\partial \phi }{\partial \eta }\right]}_{i-1/2}^{i+1/2}-{\left[\beta \frac{\Gamma_{\phi }}{J}\frac{\partial \phi }{\partial \eta }\right]}_{j-1/2}^{j+1/2}$$ (33)

Employing the central difference scheme for the diffusion fluxes and the third-order MUSCL scheme for the convection fluxes, Eqs 31 and 32 can be expressed in the following generalized iterative form

$$A_{i,j}^{\phi }\phi_{i,j}=A_{i+1}^{\phi }\phi_{i+1}+A_{i-1}^{\phi }\phi_{i-1}+A_{j+1}^{\phi }\phi_{j+1}+A_{j-1}^{\phi }\phi_{j-1}+S_{i,j}^{\phi }$$ (34)

where

$$\begin{array}{l} A_{i,j}^{\phi }=J\frac{{\tilde{\rho }}_{i,j}}{\Delta t}+A_{i+1}^{\phi }+A_{i-1}^{\phi }+A_{j+1}^{\phi }+A_{j-1}^{\phi }\\ A_{i+1}^{\phi }=D_{i+1/2}^{\phi }+\frac{F_{i+1/2}^{\phi }-[F_{i+1/2}^{\phi }]}{2},\;A_{j+1}^{\phi }=D_{j+1/2}^{\phi }+\frac{F_{j+1/2}^{\phi }-[F_{j+1/2}^{\phi }]}{2}\\ A_{i-1}^{\phi }=D_{i-1/2}^{\phi }+\frac{F_{i-1/2}^{\phi }+[F_{i-1/2}^{\phi }]}{2},\;A_{j-1}^{\phi }=D_{j-1/2}^{\phi }+\frac{F_{j-1/2}^{\phi }+(F_{j-1/2}^{\phi }]}{2} \end{array}$$ (35)

with

$$\begin{array}{l} F_{i+1/2}^{T}={\left(\tilde{\rho }U\right)}_{i+1/2},F_{i-1/2}^{\phi }={\left(\tilde{\rho }U\right)}_{i-1/2},F_{j+1/2}^{\phi }={\left(\tilde{\rho }V\right)}_{j+1/2},F_{j-1/2}^{\phi }={\left(\tilde{\rho }V\right)}_{j-1/2}\\ F_{i+1/2}^{\phi }={\left(\tilde{\rho }U\right)}_{i+1/2},F_{i-1/2}^{\phi }={\left(\tilde{\rho }U\right)}_{i-1/2},F_{j+1/2}^{\phi }={\left(\tilde{\rho }V\right)}_{j+1/2},F_{j-1/2}^{\phi }={\left(\tilde{\rho }V\right)}_{j-1/2}\\ D_{i+1/2}^{\phi }={\left(\alpha \Gamma_{\phi }/J\right)}_{i+1/2},D_{i-1/2}^{\phi }={\left(\alpha \Gamma_{\phi }/J\right)}_{i-1/2},D_{j+1/2}^{\phi }={\left(\gamma \Gamma_{\phi }/J\right)}_{j+1/2},D_{j-1/2}^{\phi }={\left(\gamma \Gamma_{\phi }/J\right)}_{j-1/2} \end{array}$$ (36)

and

$$\begin{array}{l} S_{i,j}^{T}=JS_{T}+S_{T}^{\text{non}}+S_{T}^{\text{dc}}+J{\left(\tilde{\rho }T\right)}_{i,j}^{*}/\Delta t\\ S_{i,j}^{\phi }=b_{\phi }(p_{i+1/2}-p_{i-1/2})+c_{\phi }(p_{j+1/2}-p_{j-1/2})+JS_{\phi }+S_{\phi }^{\text{non}}+S_{\phi }^{\text{dc}},\;\phi =u,v,k,\varepsilon \end{array}$$ (37)

The deferred correction for convection item $S_{\phi }^{\text{dc}}$ (*ϕ* = *u*, *v*, *k*, *ε*, *T*) is introduced by the blending of the third-order MUSCL scheme and the first-order upwind difference scheme. Due to the existence of strong shock waves in the high-speed airflow, a continuous-differential limiter with Θ = 1 × 10^−30^ is used in the third-order MUSCL scheme to eliminate the spurious numerical oscillations in shock region, defined by [39]

$$\psi_{i}=\frac{2(\phi_{i}-\phi_{i-1})(\phi_{i+1}-\phi_{i})}{{\left(\phi_{i}-\phi_{i-1}\right)}^{2}+{\left(\phi_{i+1}-\phi_{i}\right)}^{2}+\Theta }$$ (38)

In addition, the pressure equation can be obtained by combining the momentum equation and continuity equation by referring to the SIMPLEM algorithm presented by Acharya and Moukalled [40] (see S1 Appendix for details). The classical deferred correction-TDMA algorithm is employed to solve the discrete governing equations. In order to emphasize on the high-temperature oxidation behavior more effectively, a segregated fluid-solid coupling solution method is applied to calculate the heat transfer of the forced air quenching, in which the fluid flow is considered as steady-state while the heat transfer between the fluid and solid domains is considered as transient. The convergent temperature field of the steady flow will be taken as the initial iterative values of the heat transfer. And the steady pressure field and the transient temperature field of each time step will be used to update the scale thicknesses of the three oxide layers FeO, Fe_3_O_4_ and Fe_2_O_3_, as well as the grid point coordinates and the thermophysical properties accordingly. The whole iteration of heat transfer of the forced air quenching will not be terminated until the end of pre-set quenching time.

### Grid treatment for the high-temperature oxidation

**Fig 3:**
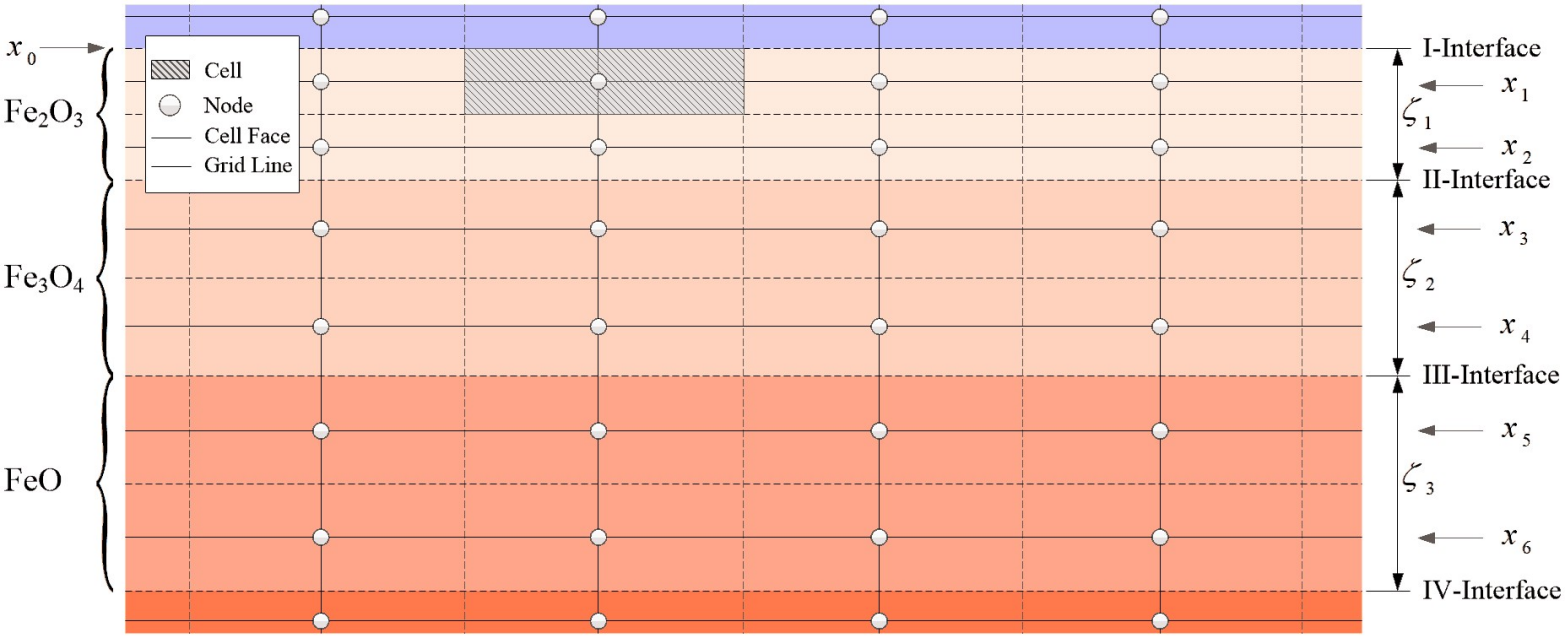
Grid treatment for the high-temperature oxidation.

In order to introduce the non-isothermal and non-uniform high-temperature oxidation, some special grid treatments need to be made to the developed model. At the beginning of the high-temperature oxidation, the surface material of the metal disk is gradually transformed into the oxide scales including the hematite, magnetite and wustite, while the scale thicknesses are continually increasing with the quenching time. To accurately reflect the heat transfer characteristics during the non-isothermal and non-uniform high-temperature oxidation, a specially designed grid treatment is provided to take into account the variations of the grid coordinates and thermophysical properties of the three oxide scales. The grid treatment for the high-temperature oxidation is shown in Fig 3.

As indicated in Fig 3, each oxide layer contains two equal cells in the axial direction. The axial coordinate of each point in the cell center varies with the increase of the scale thickness. Hence, the axial coordinate *x*_*i*_ of the *i*th set of points in the oxide scales is determined by

$$x_{i}=\{\begin{array}{l} x_{0}+\sum_{j=1}^{\left(i-1\right)/2}\zeta_{j}+\frac{1}{4}\zeta_{i},\;i=1,3,5\\ x_{0}+\sum_{j=1}^{\left(i-2\right)/2}\zeta_{j}+\frac{3}{4}\zeta_{i},\;i=2,4,6 \end{array}$$ (39)

At the beginning of the iterative solution of the heat transfer problem, the thermophysical properties in the above oxide region are set to the same values as the substrate material of the metal disk. In the subsequent iteration, the corresponding thermophysical properties are modified to the values of the three oxide scales.

## High-temperature oxidation experiment

In order to inversely identify the parabolic parameters of the developed oxidation kinetic model by the multi-objective optimization using NSGA-II, the high-temperature oxidation experiment should be implemented appropriately. The heat insulation treatment before the oxidation experiment and the anti-oxygenation treatments in heating process and subsequent cooling process after quenching are the key to the successful implementation of the high-temperature oxidation experiment of the forced air quenching.

**Fig 4:**
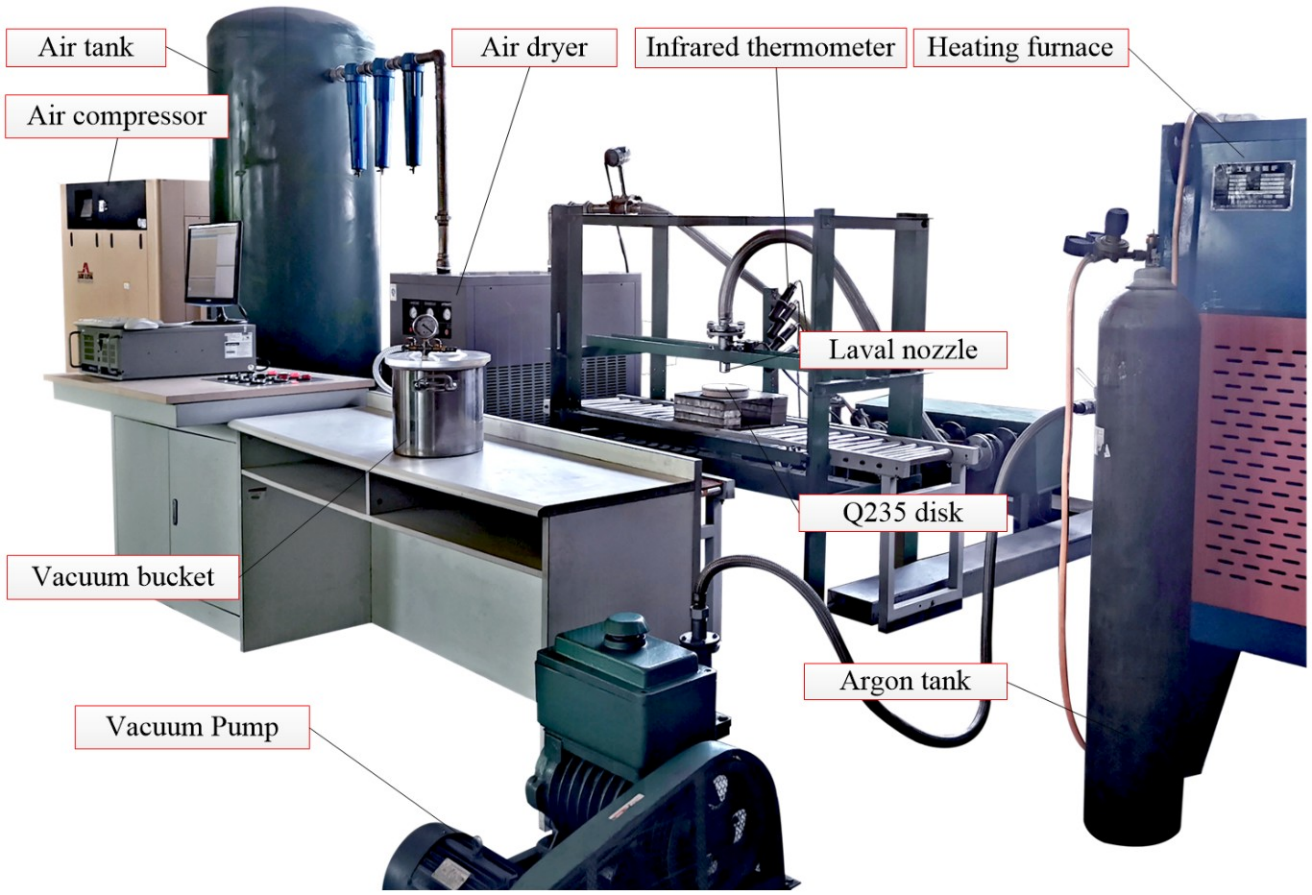
Experimental device of the high-temperature oxidation during the quenching process.

As shown in Fig 4, the experimental device of the high-temperature oxidation of the forced air quenching of a Q235 disk mainly includes the LG100 air compressor, HH-2K-25 air tank, YD-100HP air dryer, IS-1300A infrared thermometer, SX2-8-13 heating furnace, 2BW4 vacuum pump, 12L vacuum bucket and 40L argon cylinder. The auxiliary materials including the high temperature resistant insulation paint and insulation board are also used before the high-temperature oxidation experiment to achieve the adiabatic treatments of the disk.

The experimental scheme of the high-temperature oxidation mainly involves seven critical steps: firstly, spraying the high temperature resistant insulation paint onto the bottom and side of the metal disk; secondly, placing the metal disk inside the base and top insulation boards and putting them into the heating furnace; thirdly, vacuuming the heating furnace and filling the inert gas argon into the furnace, then heating the metal disk to 1173.15 K; fourth, taking out the metal disk and immediately performing the forced air quenching after removing the top insulation board, then recording the surface temperatures with the infrared thermometers; fifth, terminating the experiment when it is on the pre-set quenching time, and simultaneously putting the metal disk into the vacuum bucket; sixth, pumping it into vacuum and filling it with argon at a certain negative pressure; finally, making the micro-sections of the oxidized metal disk when it cooled to room temperature, and observing the scale thickness by the optical microscope.

**Fig 5:**
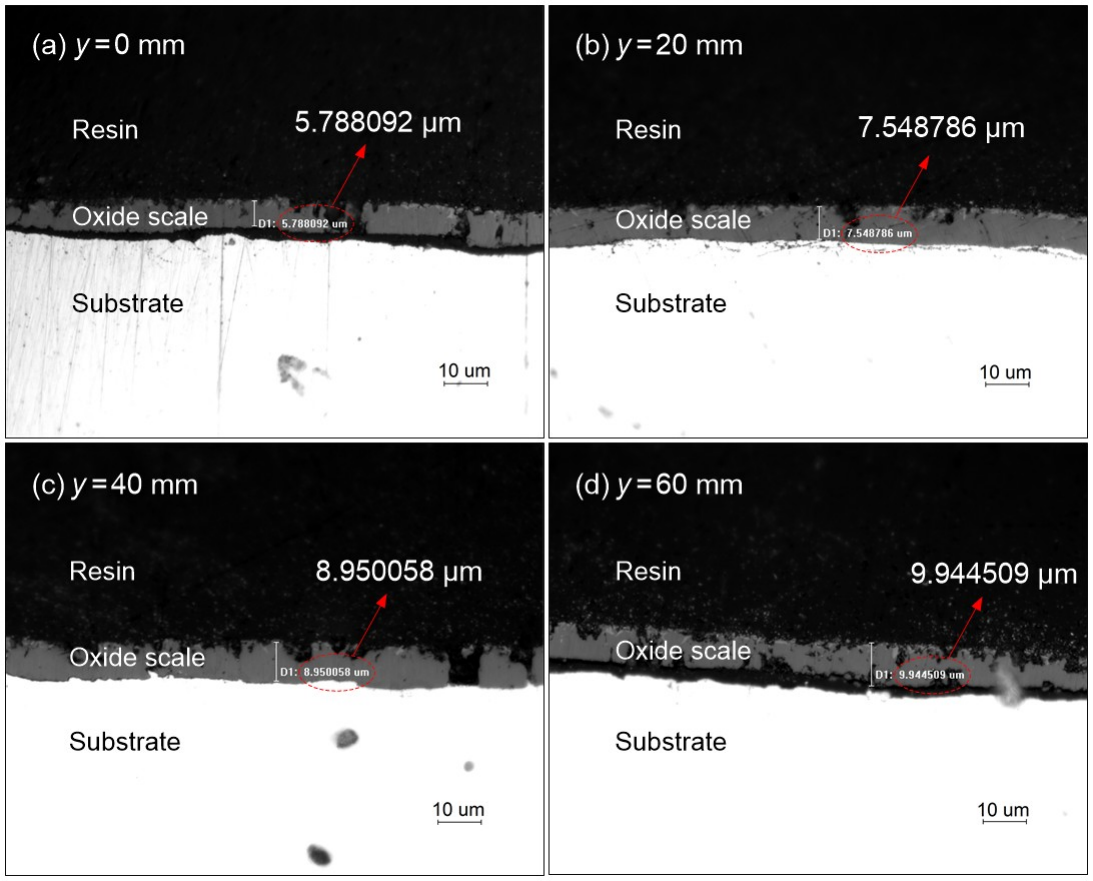
Cross-sectional microstructure of the oxide scale at different radial positions.

To get the instantaneous scale thickness for the proposed inverse identification method, the high-temperature oxidation experiment of the forced air quenching with *t* = 50 s is performed. The measured positions of the instantaneous thicknesses of the three oxide scales are arranged at *y* = 0 mm, 20 mm, 40 mm and 60 mm along the radial direction of the metal disk. However, due to the characteristics of short-time high-temperature oxidation (with thin oxide scale in micron level), it is extremely difficult to observe the respective thicknesses of the three oxide layers. Thus, in present work, only the total thickness of the oxide scale is measured. Taking the Q235 disk (*r* = 80 mm, *d* = 15 mm and *h* = 60 mm) for example, the instantaneous total scale thicknesses at different radial positions during the oxidation experiment are measured. The corresponding cross-sectional optical images of the oxide scale at different radial positions are shown in Fig 5.

## Numerical and experimental examples

**Fig 6:**
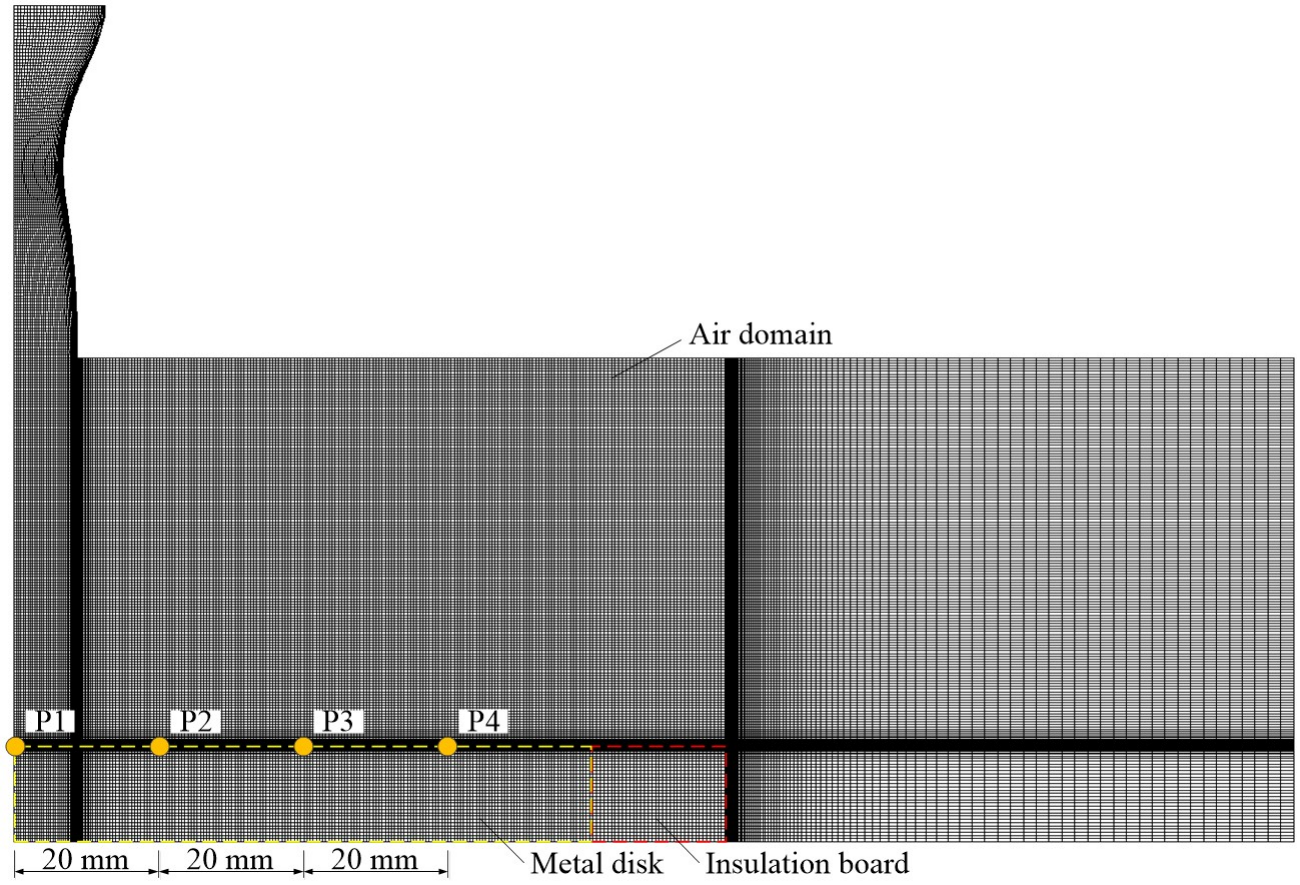
Mesh arrangement of fluid and solid domains of the forced air quenching.

In order to verify the correctness and effectiveness of the developed formulas and method, both the numerical and experimental examples of the forced air quenching of a Q235 disk are investigated. The experimental method is the same as the first seven critical steps of the high-temperature oxidation experiments of the forced air quenching. The mesh grid of the fluid and solid domains is generated by ICEM CFD, as shown in Fig 6, which contains 19126 nodes and 20064 cells. Noted that the grid independence test has been carefully made to ensure the dependability of the numerical results as prerequisite. The initial total pressure and temperature at the inlet of the Laval nozzle are set to 0.6 MPa and 298 K, respectively. The initial temperatures of the fluid domain and the solid domain are set to 298 K and 1173.15 K, respectively, and the specific heats of the two domains are 1006.3 J/(kg·K) and 460 J/(kg·K). The thermophysical properties of the three oxide layers FeO, Fe_3_O_4_ and Fe_2_O_3_ are listed in Table 1 [41, 42]. The pre-set time of the forced air quenching lasts 50 s. The developed formulas are implemented by a self-programed Fortran code, and the data post-processing is based on a self-programed MATLAB code.

**Table 1:** Thermophysical properties of the three oxide scales.

Oxide layer	Fe_2_O_3_	Fe_3_O_4_	FeO
Density (kg/m^3^)	524	518	570
Specific heat (J/kg K)	989	937	725
Thermal conductivity (W/m K)	4.9	3.7	7.5

**Fig 7:**
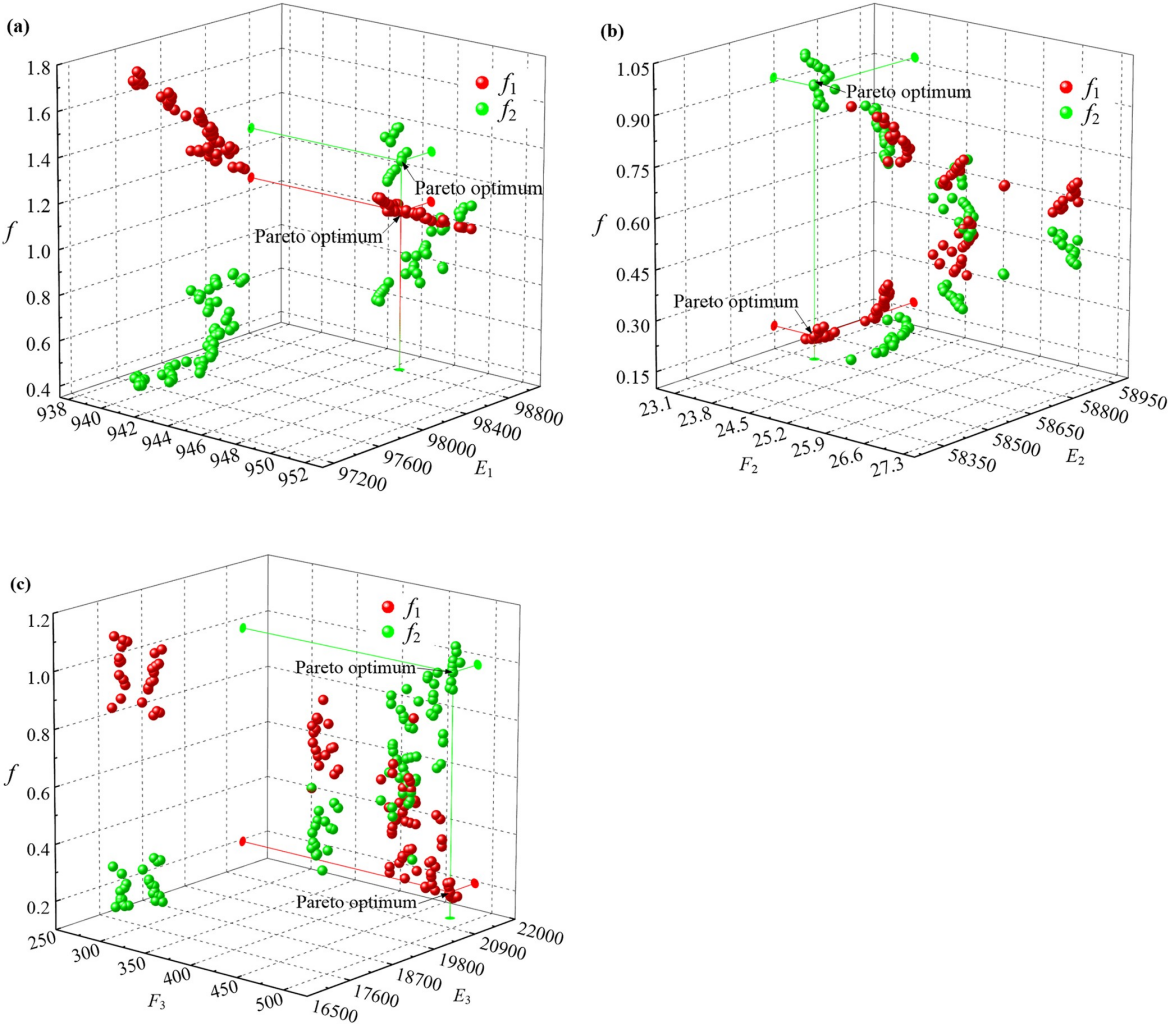
Pareto fronts of the multi-objective optimization of the parabolic parameters. (a) *E*_1_ and *F*_1_, (b) *E*_2_ and *F*_2_, (c) *E*_3_ and *F*_3_.

Based on the measured instantaneous thicknesses of the oxide scales, the parabolic parameters of the developed oxidation kinetic model are identified by the multi-objective optimization algorithm using NSGA-II with *w*_*l*_ = 1(*l* = 1, 2, 3, 4), and the corresponding Pareto fronts of the multi-objective optimization of the parabolic parameters are shown in Fig 7. The Pareto optimums are obtained by applying the evaluation function *h*(*F*_*l*_) with weighting coefficients ${\tilde{w}}_{1}=0.9$ and ${\tilde{w}}_{2}=0.1$. The identified values of the parabolic parameters are listed in Table 2.

**Table 2:** Identified parabolic parameters of the developed oxidation kinetic model.

Parameters	*E*_1_	*E*_2_	*E*_3_	*F*_1_	*F*_2_	*F*_3_
Identified values	98816.05	58648.22	21329.61	946.51	23.52	481.27

## Results and discussion

### Model verification

**Fig 8:**
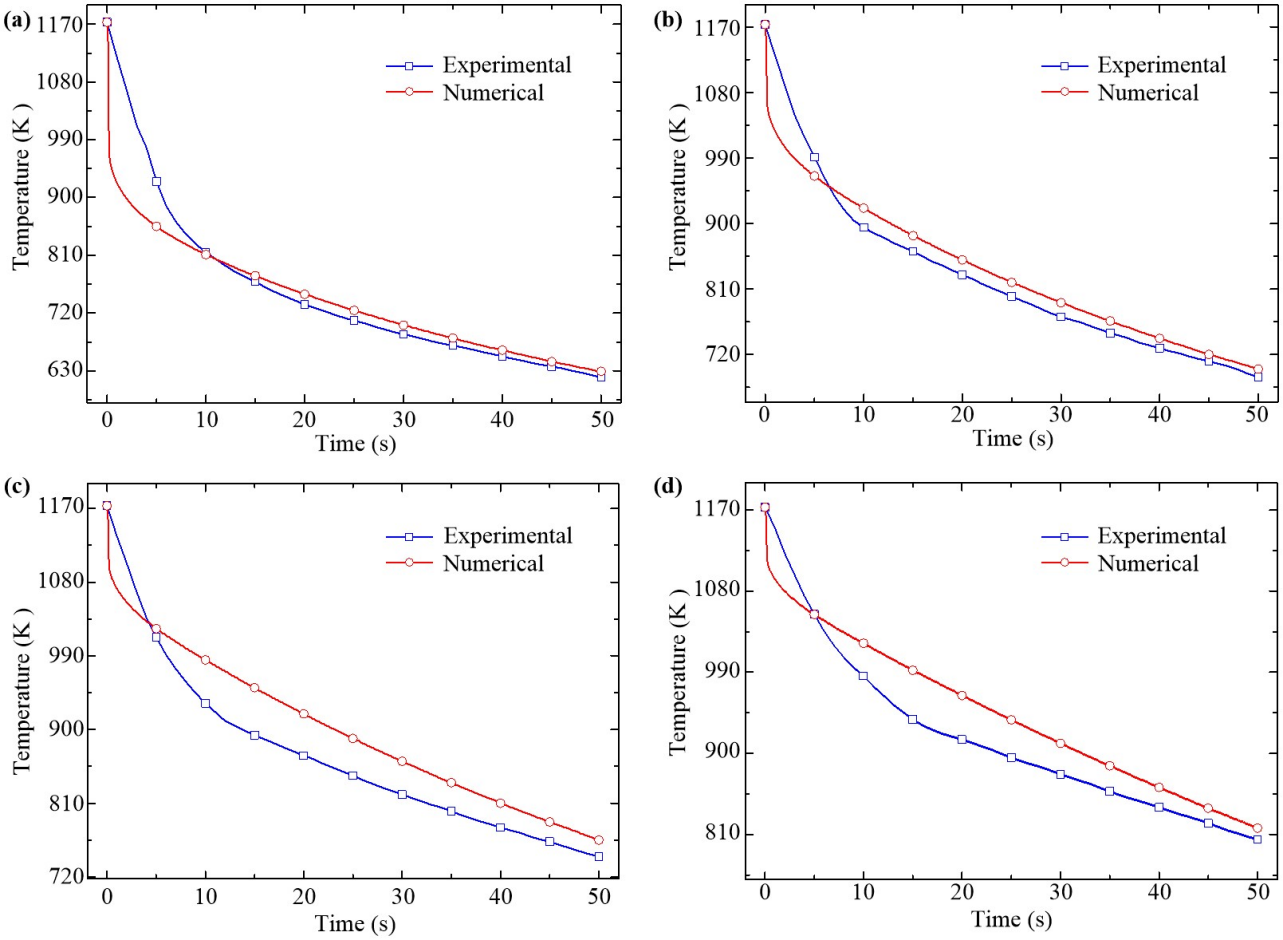
Comparisons of numerical and experimental temperatures at different radial positions. (a) *y* = 0 mm, (b) *y* = 20 mm, (c) *y* = 40 mm, (d) *y* = 60 mm.

Taking the radial positions of *y* = 0 mm, 20 mm, 40 mm and 60 mm at the conjugate interface for example, the comparison between the numerical and experimental temperature drops of the four representative points in 50 s are shown in Fig 8. It is evident from Fig 8 that the numerical temperature drops calculated by the developed formulation are well consistent with the experimental results observed in present study, with acceptable relative deviations of 8.80%, 5.37%, 7.10%, 11.64%, respectively, which validate the correctness and effectiveness of the developed formulas for heat transfer of the forced air quenching with considering the non-isothermal and non-uniform high-temperature oxidation.

**Fig 9:**
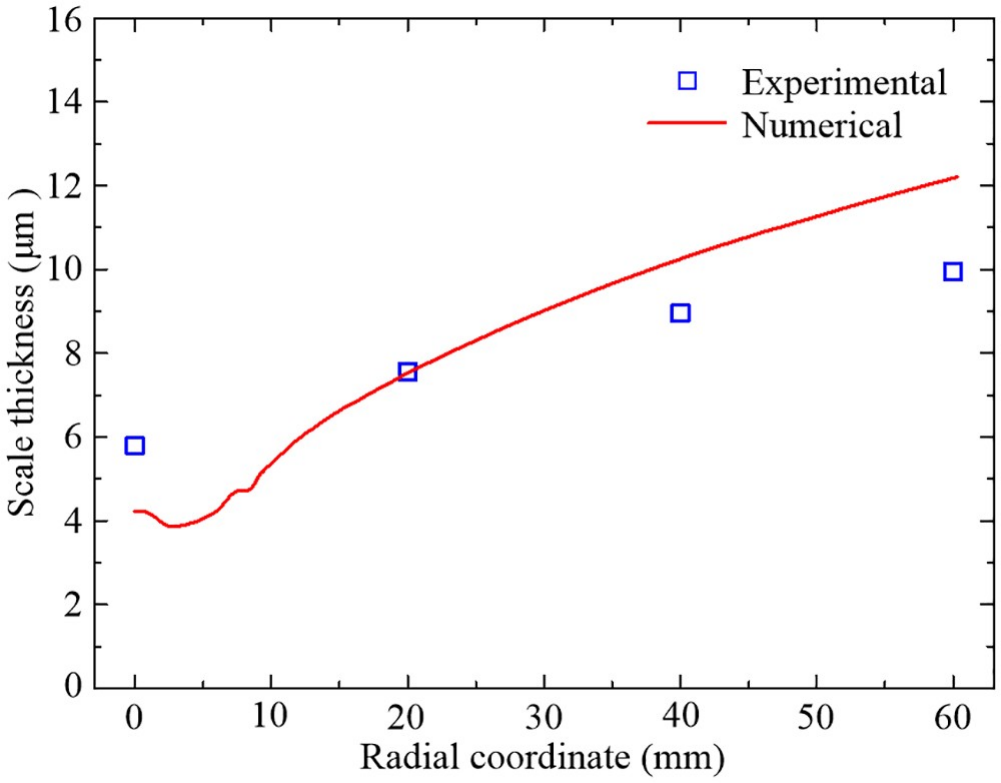
Comparisons of numerical and experimental scale thicknesses.

Moreover, further comparison is made between the predicted and measured scale thicknesses at 50 s to investigate the applicability of the developed oxidation kinetic model, as shown in Fig 9. Results indicate that the predicted scale thicknesses at the four representative points at 50 s basically agree with the measured data in high-temperature oxidation experiment while the relative deviations are 27.21%, 0.78%, 13.50% and 22.69%, respectively. Considering the submicron level scale thickness, the highly nonlinear oxidation behavior and the complex turbulent flow conditions, it is still perfectly acceptable for the numerical results with the maximum relative deviation under 30%, which validates the feasibility of the developed oxidation kinetic model and corresponding parameter identification method in dealing with high-temperature oxidation behavior in the forced air quenching process.

Nevertheless, some differences still inevitably exist between the numerical and experimental results. The reasons can be summarized as follows: (1) The employed realizable *k*-*ε* turbulence model and wall functions are still semi-empirical, some treatments may not fully adapt to deal with the problems of the forced air quenching, which can result in a certain temperature deviation; (2) Due to the temperature and oxygen partial pressure dependences of the growth of the oxide scale, and even the temperature dependence of the oxygen partial pressure, the temperature deviation would further increase the thickness deviation of the numerical oxide scale; (3) In the actual operation of the scale thickness measurement, the preparation of metallography including the processes of the cutting, inlaying, rough grinding, fine grinding, polishing, corrosion, and so on, should be implemented. The complexity of the preparation process may cause a certain thickness deviation of the measured oxide scale.

### Heat transfer characteristics analysis

**Fig 10:**
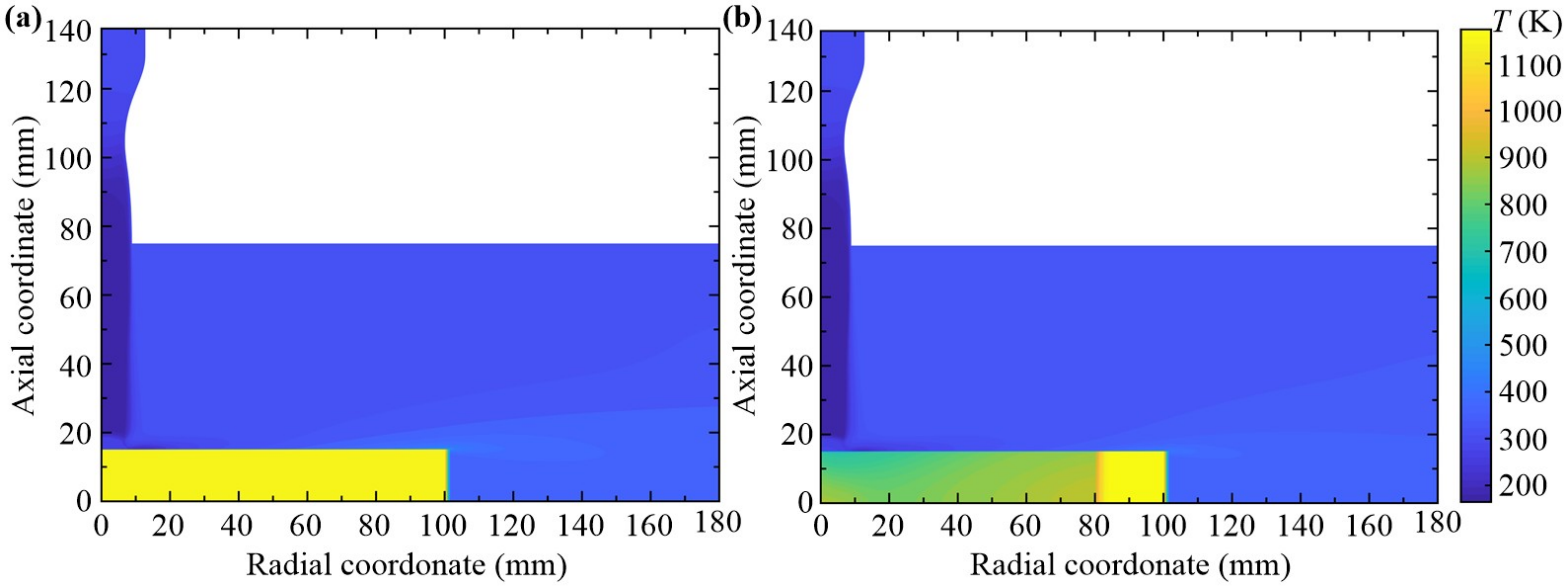
Temperature field distributions of the forced air quenching. (a) Steady-state flow, (b) Heat transfer at 50 s.

**Fig 11:**
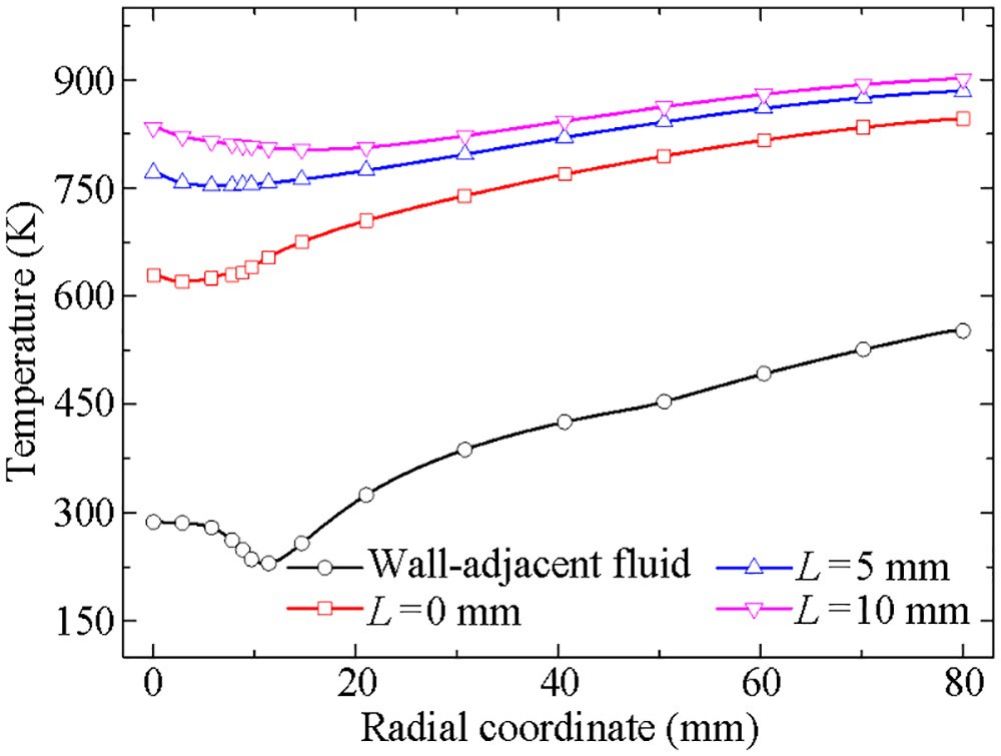
Comparison of the temperature distributions of the defined three different radial paths.

The temperature field distributions of the steady-state flow and the heat transfer at 50 s are shown in Fig 10. The temperature field of the steady-state flow is taken as the initial condition for heat transfer analysis while other flow parameters are considered constant. From Fig 10 we can see that the inlet airflow is generally transformed into the low-temperature airflow, and recuperates in the stagnation region. In the radial direction of the wall-adjacent region, the temperature firstly decreases and then increases with the increasing of the radial coordinate. This distribution law of the steady-state temperature field has a great extent to determine the heat transfer characteristic of the forced air quenching, which can be better illustrated in Fig 11.

As we can see from Fig 11, a comparison between the temperature distributions of the four radial paths (one is the wall-adjacent fluid, the others are the three radial paths *L* = 0 mm, 5 mm and 10 mm from the top surface of the disk) is made. The results indicate that not only the convection heat transfer plays a dominant part in the conjugate temperature distribution, but also the heat conduction predominates over other factors inside the disk, and makes the inner temperature more uniform to some extent.

**Fig 12:**
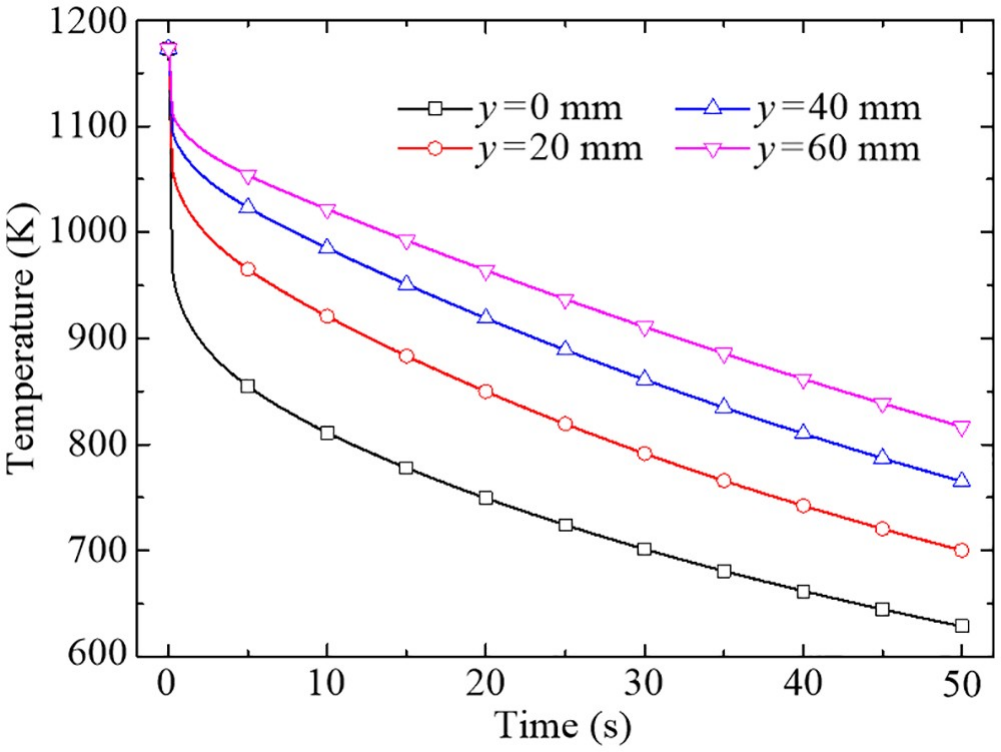
Temperature drops of the four representative points at the conjugate interface in 50 s.

In order to demonstrate the discipline of transient temperature variation at the conjugate interface, the temperature drops at the four representative points in 50 s are shown in Fig 12. Generally, the temperature decreases more and more slowly over the quenching time. This is largely due to the initial temperature difference between the wall-adjacent fluid and the top surface of the disk. As the temperature difference decreases in the quenching process, the heat flux at the conjugate interface also continues to decrease with time, eventually leading to a gradual decrease of the temperature drop rate. However, it’s worth mentioning that the average temperature drop rate of the four representative points in the first 20 seconds is up to 21.19 K/s, 16.16 K/s, 12.71 K/s and 10.45 K/s, respectively, which happens to be at the phase transition temperature region. The results indicate that the temperature drop rate in the stagnation region is larger than other positions at the conjugate interface during the quenching process as a whole, further illustrate the active quenching region of the forced air quenching is mainly centralized in the vicinity of stagnation region.

**Fig 13:**
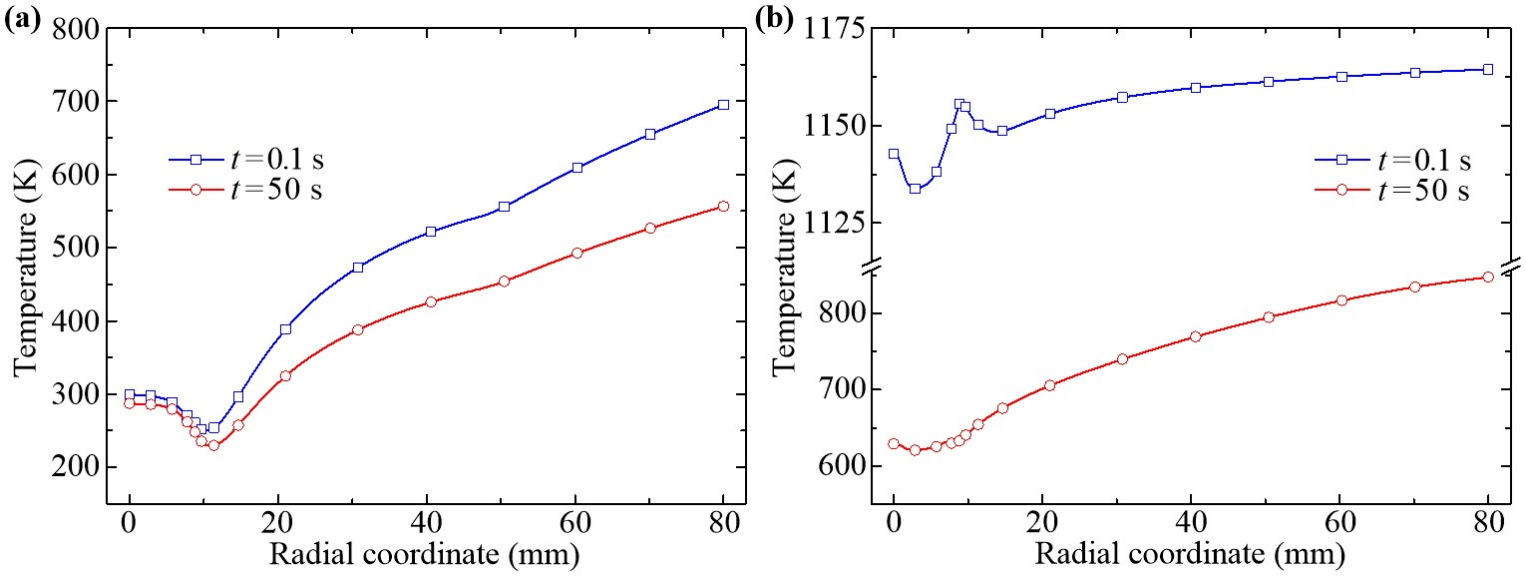
Comparisons of the radial temperature distributions at time 0.1 s and 50 s. (a) Wall-adjacent fluid, (b) Disk upper-wall.

Moreover, the temperature distributions of the wall-adjacent fluid and the upper-wall solid along the radial direction at 0.1 s and 50 s are shown in Fig 13. Here the time 0.1 s approximately represents the beginning phase of the forced air quenching. As can be seen from Fig 13, the temperature distribution rules of the wall-adjacent fluid during the quenching process are basically the same while the temperature values only have a slight decrease from 0.1 s to 50 s. The degree of decrease mainly depends on the employed wall functions and the upper-wall temperature of the disk. In addition, it also shows that the temperature distribution of the upper-wall solid at 0.1 s is largely determined by the temperature distribution of the wall-adjacent fluid, that is, by the intense turbulent convection heat transfer. In contrast, the temperature values of the upper-wall solid at 50 s appear to drop considerably, while the temperature distribution becomes more uniform due to the inner heat conduction of the disk.

**Fig 14:**
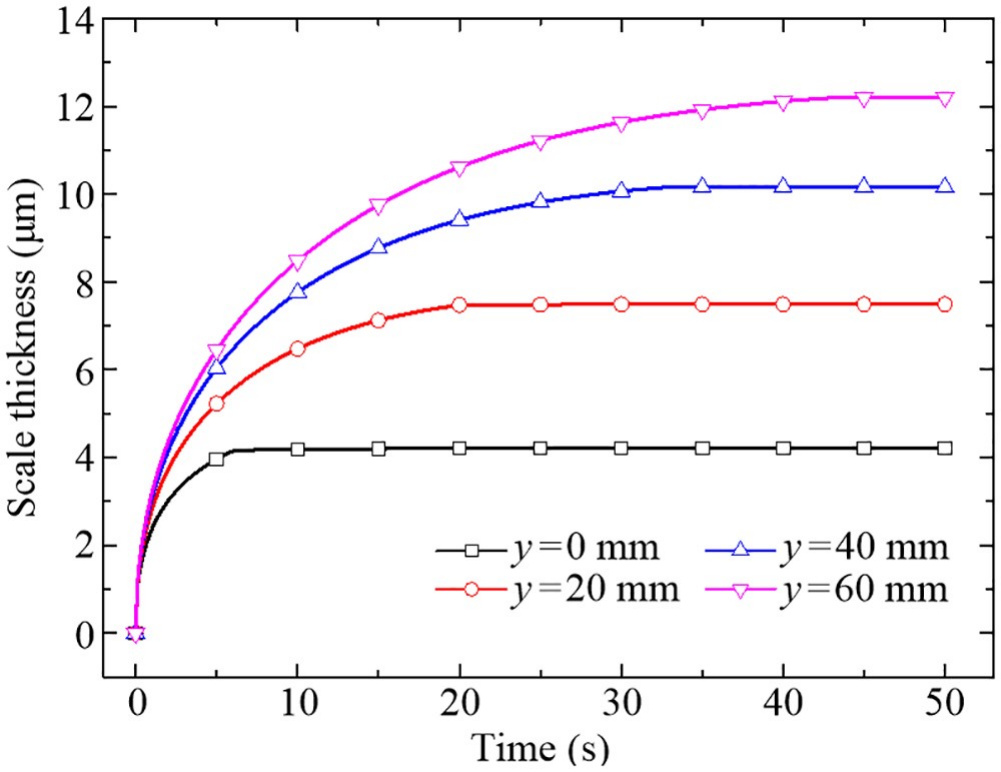
Variations of scale thickness of the four representative points in 50 s.

The variations of scale thicknesses at the four representative points during the quenching process are shown in Fig 14. As you can see from Fig 14, the scale thickness at *y* = 0 mm increases logarithmically in the first 5 s and then remains roughly the same for the rest of the quenching time. Similarly, the scale thicknesses at *y* = 20 mm, 40 mm and 60 mm reach a relatively stable state at about 20 s, 30 s and 40 s, respectively. The stable state is actually achieved by the non-growth mechanism of the layer of FeO below 843.15 K which has been carefully considered in the developed oxidation kinetic model. In general, the higher the temperature is, the faster the oxidation reaction rate will be. This pattern happens to reflect the growth characteristics of the oxide scales with temperature, as indicated in Figs 12 and 14, and further results in the similar distribution law of scale thickness along the radial direction are shown in Fig 9. The growth of the oxide scale will form a certain thermal resistance at the conjugate interface, which in turn influence the heat transfer between the fluid and solid domains.

**Fig 15:**
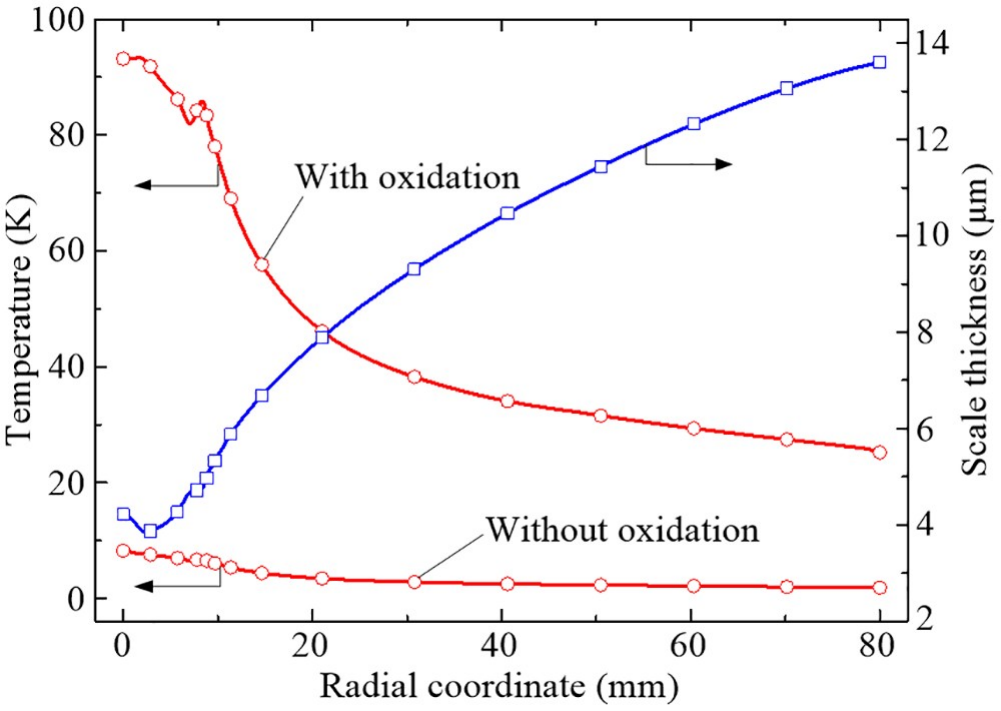
Distributions of scale thickness and temperature difference of the total oxide layer.

To illustrate the effects of the growth of the oxide scale on the heat transfer characteristics of the forced air quenching, the temperature difference across the total oxide layer, the transient and radial temperature variations in specific oxide interface with and without oxidation are analyzed. Corresponding to the scale thickness, the radial distributions of the temperature difference across the total oxide layer with and without oxidation are given in Fig 15.

From Fig 15 we can see that the high-temperature oxidation has great effects on the temperature difference across the total oxide layer, especially in the stagnation region. Furthermore, the variation regularity of the temperature difference with the radial coordinate is the opposite to that of the scale thickness, which seems to be that thinner oxide scale may be in some sense more benefit for heat transfer across the whole oxide layer. Indeed, the conclusion can be easily obtained by using Newton’s law of cooling, that is, under the condition of constant heat flux density, the smaller the thermal conductivity, the larger the temperature gradient. According to the differential form of the Newton’s law of cooling, it can be concluded that the radial variation regularity of the temperature difference is mainly determined by the thermal conductivity and the scale thickness of the total oxide layer.

## Conclusions

In this paper, a finite volume model of heat transfer of the forced air quenching with considering the non-isothermal and non-uniform oxidation is established, in which a three-layered non-isothermal high-temperature oxidation kinetic model is developed by introducing the variations of interfacial temperature and oxygen partial pressure along the radial direction. And a parameter identification method using the multi-objective genetic algorithm is proposed for the inverse solution of the oxidation parabolic parameters based on the measured scale thicknesses in oxidation experiments. The numerical results of interfacial temperature and scale thickness show a good agreement with experimental data, and the relative deviations are within 11.64% and 27.21%, respectively, are perfectly acceptable by considering the submicron level scale thickness, the highly nonlinear oxidation behavior and the complex turbulent flow conditions.

Furthermore, the heat transfer characteristics and the effects of the high-temperature oxidation are analyzed. Results indicate that the interfacial temperature distribution of the steady-state temperature field has a great extent to determine the heat transfer characteristic, and not only the convection heat transfer plays a dominant part in the conjugate temperature distribution, but also the heat conduction predominates over other factors inside the disk, and makes the inner temperature more uniform to some extent. The temperature drop rate in the stagnation region is larger than other positions at the conjugate interface during the quenching process as a whole, which further illustrate that the active quenching region is mainly centralized in the vicinity of stagnation region. In addition, the scale thickness in early stage increases logarithmically with time and then remains roughly the same until reaching the non-growth condition of the layer of FeO. Moreover, the high-temperature oxidation has great effects on the temperature difference across the total oxide layer, especially in the stagnation region. The radial variation regularity of the temperature difference is mainly determined by the thermal conductivity and the scale thickness of the total oxide layer. The existence of the oxide scale actually produces a certain thermal resistance during the quenching process and the effects of the oxide scale increases with the radial coordinate due to the interfacial temperature distribution. The results obtained can provide theoretical derivation for precise control of the internal phase transformation during the quenching process.

## Supporting information

S1 Appendix Pressure equation. (DOCX)
