# Correction: Heat transfer analysis of the forced air quenching with non-isothermal and non-uniform oxidation

**DOI:** 10.1371/journal.pone.0270448

**Published:** 2022-06-21

**Authors:** The PLOS ONE Staff

The fourth author’s name is spelled incorrectly. The correct name is: Hua Song. The correct citation is: Zhang Y, Yang J, Gao M-X, Song H (2021) Heat transfer analysis of the forced air quenching with non-isothermal and non-uniform oxidation. PLoS ONE 16(6): e0253240. https://doi.org/10.1371/journal.pone.0253240. The publisher apologizes for the error.
