# Supplementary material for: Heat transfer analysis of the forced air quenching with non-isothermal and non-uniform oxidation
Source: PLoS One. 2021 Jun 17;16(6):e0253240. doi: 10.1371/journal.pone.0253240 (PMC8211215; doi:10.1371/journal.pone.0253240)
Supplement: S1 Appendix — (DOCX) [file pone.0253240.s001.docx]

**S1 Appendix**

Yue Zhang, Jian Yang, Ming-Xin Gao, Hua Song

**Pressure equation**

The pressure equation can be obtained by combining the momentum equation and continuity equation, given by

$A_{i,j}^{p}p_{i,j}=A_{i+1}^{p}p_{i+1}+A_{i-1}^{p}p_{i-1}+A_{j+1}^{P}p_{j+1}+A_{j-1}^{p}p_{j-1}+S_{i,j}^{p}$ (1)

where

$\begin{matrix} A_{i,j}^{p}=A_{i+1}^{p}+A_{i-1}^{p}+-A_{j+1}^{p}+A_{j-1}^{p} \\ A_{i+1}^{p}\text{=}-\left(\rho B_{u}\right)_{i+1/2}, A_{i-1}^{p}\text{=}-\left(\rho B_{u}\right)_{i-1/2} \\ A_{j+1}^{p}\text{=}-\left(\rho C_{v}\right)_{j+1/2}, A_{j-1}^{p}\text{=}-\left(\rho C_{v}\right)_{j-1/2} \end{matrix}$ (2)

with

$\begin{matrix} B_{u}=\left(b_{u}y_{\eta}-b_{v}x_{\eta}\right)/A_{i,j}^{u}, C_{u}=\left(c_{u}y_{\eta}-c_{v}x_{\eta}\right)/A_{i,j}^{u} \\ B_{v}=\left(b_{v}x_{\xi}-b_{u}y_{\xi}\right)/A_{i,j}^{v}, C_{v}=\left(c_{v}x_{\xi}-c_{u}y_{\xi}\right)/A_{i,j}^{v} \end{matrix}$ (3)

The source item for the pressure equation is determined by

$S_{i,j}^{p}=\hat{S}_{i,j}^{p}+S_{p}^{\text{non}}+S_{p}^{\text{axis}}$ (4)

where

$\begin{matrix} S_{p}^{\text{axis}}=-\left(J\rho v/y\right)_{i,j} \\ \hat{S}_{i,j}^{p}=-\left[\left(\rho\hat{U}\right)_{i+1/2}-\left(\rho\hat{U}\right)_{i-1/2}+\left(\rho\hat{V}\right)_{j+1/2}-\left(\rho\hat{V}\right)_{j-1/2}\right] \\ S_{p}^{\text{non}}=-\left[\left(\rho C_{u}p_{\eta}\right)_{i+1/2}-\left(\rho C_{u}p_{\eta}\right)_{i-1/2}+\left(\rho B_{v}p_{\xi}\right)_{j+1/2}-\left(\rho B_{v}p_{\xi}\right)_{j-1/2}\right] \end{matrix}$ (5)

where $\hat{U}$ and $\hat{V}$ refer to the pseudo-contravariant velocity components, which are calculated by the pseudo-velocity components with removing the pressure terms in the momentum equations.
